# Chromatin and aberrant enhancer activity in *KMT2A* rearranged acute lymphoblastic leukemia

**DOI:** 10.1016/j.gde.2024.102191

**Published:** 2024-04-04

**Authors:** Thomas A Milne

**Affiliations:** MRC Molecular Haematology Unit, MRC Weatherall Institute of Molecular Medicine, Radcliffe Department of Medicine, University of Oxford, Oxford OX3 9DS, UK

## Abstract

To make a multicellular organism, genes need to be transcribed at the right developmental stages and in the right tissues. DNA sequences termed ‘enhancers’ are crucial to achieve this. Despite concerted efforts, the exact mechanisms of enhancer activity remain elusive. *Mixed lineage leukemia* (*MLL* or *KMT2A*) rearrangements (MLLr), commonly observed in cases of acute lymphoblastic leukemia (ALL) and acute myeloid leukemia, produce novel in-frame fusion proteins. Recent work has shown that the MLL-AF4 fusion protein drives aberrant enhancer activity at key oncogenes in ALL, dependent on the continued presence of MLL-AF4 complex components. As well as providing some general insights into enhancer function, these observations may also provide an explanation for transcriptional heterogeneity observed in MLLr patients.

## Introduction

In 1981, it was shown that SV40 viral sequences could massively increase transcription of the β-globin gene in transient transfection assays [1]. Since then, the general importance of ‘enhancer’ sequences has been recognized as they are essential for normal tissue and cell type–-specific gene expression patterns [2–4] and when mutated can be major drivers of human disease [5]. Despite an immense amount of work over the decades, exactly how enhancers function is still not clearly understood.

However, one clear action of enhancers is to help assemble coactivator complexes. There is no precise role for a coactivator, but many coactivators are chromatin remodelers or chromatin-modifying enzymes. The basic subunit of chromatin is the nucleosome, which consists of DNA wrapped around an octamer of histone proteins, most commonly consisting of two copies each of H2A, H2B, H3 and H4. The nucleosome can act as a barrier to transcription, but the histones can also be post-translationally modified to carry specific ‘marks’ such as acetylation or methylation. A great number of chromatin proteins that have been implicated in human disease have also turned out to be coactivators or corepressors that are involved in ‘writing’, ‘erasing’ or ‘reading’ histone modifications [6].

In general, active enhancers display characteristics that include an open chromatin conformation, binding of DNA sequence–specific transcription factors, expression of short transcripts called enhancer RNAs (eRNAs), and post-translational histone modifications, such as histone H3 lysine 4 monomethylation (H3K4me1) and H3 lysine 27 acetylation (H3K27ac) [2,3]. Alongside the more commonly studied effect of DNA mutations, aberrant chromatin changes can also be a major driver of altered enhancer function and hence gene expression in human disease [5,7,8]. This review will present a brief overview of enhancer function and then will focus on some of the recent work on altered enhancer activity in the subset of specific leukemias caused by rearrangements of the *Mixed Lineage Leukemia* (*MLL* or *KMT2A*) gene.

## Protein complex formation at enhancers

One important activity of an enhancer is to act as a docking site for transcription factors, which can then activate appropriate target genes over 10 s–1000 s kb distances. A single enhancer is able to act on multiple promoters at the same time, leading to a proposed ‘hub model’ for enhancer activity, whereby enhancers function by creating localized concentrations of transcription factors, chromatin-associated proteins and coactivators in the proximity of promoters [9,10]. This hub model is consistent with previously proposed ‘transcription factories’ [11], as well as more recent models of liquid–liquid–phase separation (LLPS) condensates formed by weak molecular interactions driven by intrinsically disordered domains [12]. It is becoming increasingly recognized that many transcription factors and coactivators contain these intrinsically disordered domains, which are predicted to have no structure in isolation but are thought to interact with one another nonspecifically to drive clustering of factors.

Although condensate formation remains a popular model for the stable assembly of activation complexes at enhancers, it has been pointed out that LLPS has rarely been properly quantified *in vivo* and instead relies mainly on qualitative observations [13]. Although these observations can be consistent with LLPS, the appearance of speckles of protein concentration in the nucleus is also explained by other mechanisms, such as the localization of proteins via high-density nonspecific weak interactions with DNA [14]. In addition, in some situations, excessive LLPS condensate formation correlates with loss of complex binding to chromatin or even with transcriptional repression, suggesting that if LLPS is a major mechanism for enhancer function, there may be a concentration ‘sweet spot’ for condensate formation leading to productive transcription events [15–17]. This is not to suggest that condensate formation does not occur, but it may be that its role in transcriptional activation and enhancer function has been overestimated. Even so, the most appealing aspect of the LLPS model is the way it explains how weak molecular interactions can drive specificity and create feedback loops that stabilize the presence of specific complexes. This is a concept it shares with multivalency models for chromatin complex assembly, where multiple weakly interacting chromatin domains synergize to create specific and stable complex assembly [18]. This relationship between multivalency and LLPS formation is highlighted by recent attempts to simulate higher order chromatin structure using attributes of both concepts [19].

## Looping and enhancer proximity

Most models of enhancer function posit that it must loop out intervening DNA to come close to the target gene promoter [3,4]. Whether or not enhancer–promoter proximity is itself important for enhancer function is an area of active debate, although it is possible some of the controversies are due to technical limitations that have yet to be resolved (for a recent extensive review of different enhancer models and the technical limitations of the imaging or chromosome conformation capture [3C] approaches used to visualize loops, please see Ref. [4]). Interestingly, a recent live imaging study (in mouse ES cells) tracking the formation of large transcriptional ‘condensates’ or ‘speckles’ found that condensate proximity was a much better predictor of increased *Sox2* transcriptional bursting than enhancer proximity was [20]. Instead, enhancer deletions drastically reduced condensate formation as well as burst size and frequency, suggesting the role of the enhancer was to stabilize condensate formation in the proximity of the gene [20]. Despite observations such as this, proximity is still a useful marker of enhancer activity that allows for the identification of specific enhancer–promoter regulatory units, and chromosome conformation capture (3C) techniques are essential for this work. In particular, the new generation of high-resolution 3C methods such as Micro Capture C and Region Capture Micro-C are capable of interrogating enhancer–promoter interactions at much higher resolution than before [21–24].

**Figure 1:**
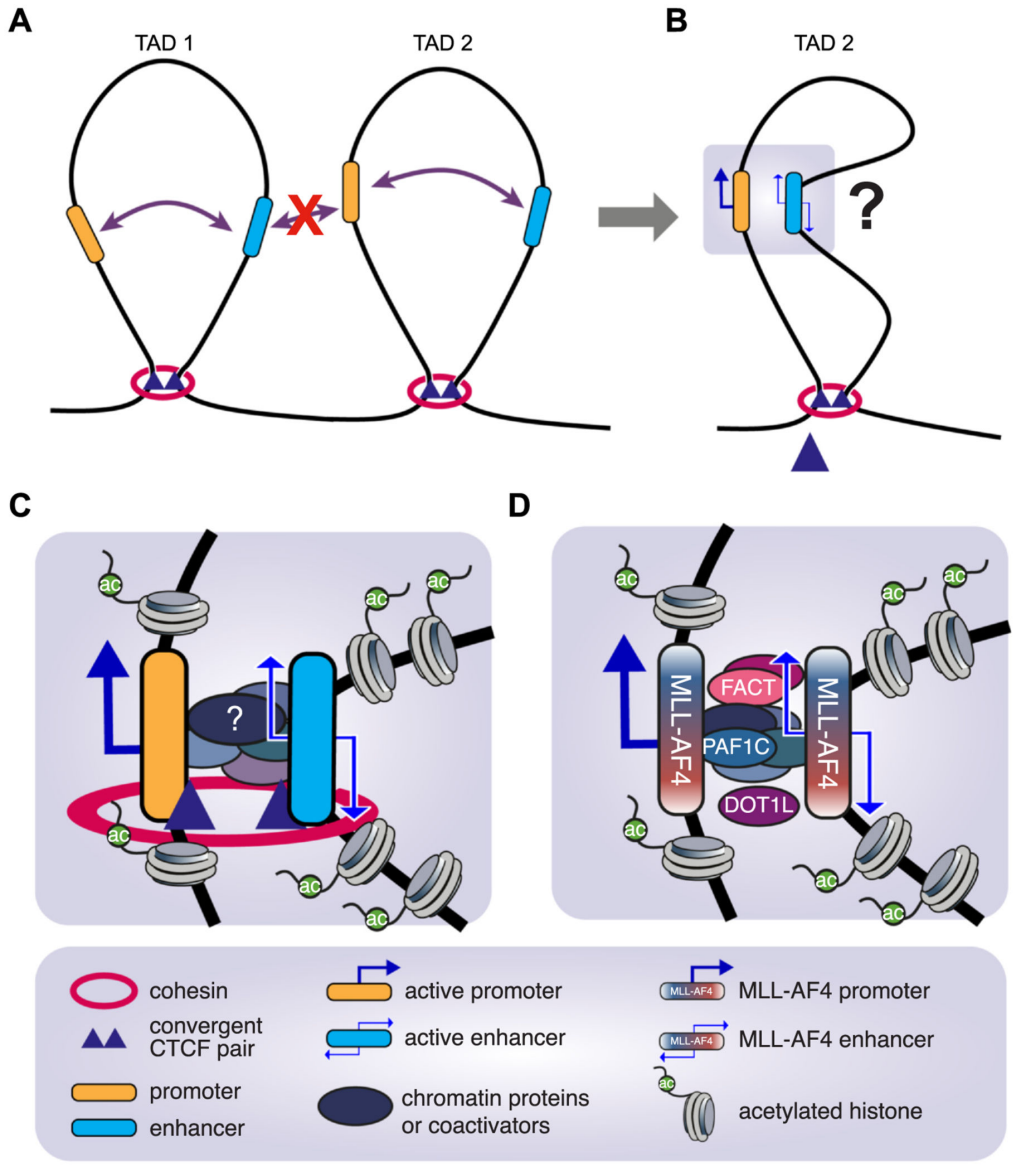
Models for enhancer function and factors that drive enhancer-promoter proximity. **(a)** The combined action of cohesin and convergent CTCF sites creates looped-out bounded domains, also known as TADs. In this example, the enhancer–promoter pairs in TAD 1 and TAD 2 have the capacity of interacting with each other, but the enhancer in TAD 1 cannot interact with the promoter in TAD 2. **(b)** Once an enhancer–promoter pair become activated within a TAD, due to unknown mechanisms, they usually come within close proximity of each other. **(c)** Several mechanisms have been proposed to drive enhancer–promoter proximity, including the binding of coactivators or chromatin proteins, or the activity of cohesin/CTCF. **(d)** At MLL-AF4 bound enhancers, enhancer–promoter proximity is driven by the assembly of a large MLL-AF4 complex that includes FACT, PAF1C and DOT1L. CTCF; CCCTC-binding factor.

A common model is that loop extrusion by the cohesin complex loops out DNA and forms structures termed topologically associating domains (TADs), which incorporate enhancers and their target gene(s) (Figure 1a). TAD boundaries are anchored by convergent CCCTC-binding factor-binding sites, which act as boundaries for cohesin and restrict enhancer–promoter interactions to those within a single TAD (Figure 1a,b). Supporting this basic model, deletion of upstream CTCF-binding sites at the α-globin locus creates an expanded sub-TAD, causing aberrant activation of nearby genes specifically in erythroid cells [3]. However, recent work has shown that cohesin/CTCF-mediated looping is both rare and dynamic, indicating that TADs are not stable structures, at least in the mouse embryonic stem cells (mESCs) where this work was done [25,26].

The mechanisms that control enhancer–promoter interactions within a TAD remain unclear. One possibility is that it is the same action of cohesin moving through the TAD that brings the enhancer and promoter within close proximity (Figure 1c). A clear example where CTCF/cohesin binding is essential for the maintenance of enhancer–promoter interactions as well as gene expression is in the acute lymphoblastic leukemia (ALL) SEM cell line at the key oncogene *MYC* [27]. Cohesin also has a role in coordinating enhancer–promoter burst frequency at inducible innate immune genes in macrophages, thus increasing the fraction of cells displaying a transcriptional burst [28]. Overall though, acute degradation of cohesin or CTCF has only minimal impacts on gene expression globally [29–32], making it unclear if cohesin has a more general role in enhancer function. One possibility is that cohesin-mediated loop extrusion may have a role in establishing enhancer–promoter proximity for high levels of transcription, and once established, basal-level, stable gene expression patterns no longer require cohesin-mediated looping [20,28].

Interestingly, depletion of CTCF, WAPL (required to release cohesin from DNA) and then cohesin results in a reorganization of higher order genome structure in mESCs, mediated by interactions between active genes [33]. Interactions between active transcriptional units also drives structural interactions in bacteria, suggesting this may be a general principle of higher order genome organization [34]. Together, these results point to the possibility that interactions between coactivator/chromatin complexes assembled on enhancers and promoters themselves drive enhancer–promoter (as well as enhancer–enhancer) interactions (Figure 1c). However, degradation of the acetylated histone reader Bromodomain-containing protein 4 (BRD4; considered to be a key contributor to condensate formation at enhancers [35,36]) has only a minimal impact on enhancer–promoter interactions despite disrupting gene expression in the leukemia SEM cell line [37]. Similarly, degradation of the coactivator mediator complex has a detectable but only subtle impact on enhancer–promoter interactions [38]. Thus, it is unclear if most coactivators have a general role in driving enhancer–promoter interactions.

Taken together, these results suggest that much remains to be understood about enhancer–promoter cross-talk, but it seems possible that different genes may depend on unique strategies for their activation. Thus, the requirement for specific protein complexes at enhancers could be unique to different enhancer–promoter units.

## Aberrant enhancers in human disease

Aberrant enhancer activity can be a major driver of human disease. For example, a novel single-nucleotide polymorphism generates a new transcription factor binding site that increases endogenous enhancer activity of the *LZTFL1* gene, resulting in increased susceptibility to coronavirus disease 2019 [22]. DNA insertion mutations can also create aberrant enhancers, such as the insertion mutations that create a novel MYB binding site upstream of the *TAL1* gene and create a super-enhancer that drives T-ALL [39]. Genome alterations can also place enhancers in close proximity to genes they do not normally regulate. One key example includes large chromosomal rearrangements that place *MYC* under the control of the immunoglobulin locus enhancers, driving poor prognosis in multiple myeloma [40]. In addition, changes in DNA methylation at enhancers are associated with diseases, such as multiple sclerosis and Alzheimer’s disease [5]. For the rest of this review, we will focus on a specific leukemia for a discussion of the significance of aberrant enhancer activity, but please see Ref. [5] for a more general discussion of enhancers in disease.

## MLL-AF4 leukemias and aberrant enhancer function

*MLL* (a.k.a. *KMT2A*) gene *r*earrangements (referred to as *MLLr*) are a major cause of poor prognosis acute myeloid leukemia (AML) or ALL, making them an unmet clinical need. MLLr are responsible for 2%–5% of childhood ALL, 5%–10% of AML and up to 70% of infant ALL [41,42]. The most common MLLr is a translocation event that fuses the *MLL* and *AF4* (a.k.a. *AFF1*) genes [43], creating novel MLL-AF4 and AF4-MLL fusion proteins. MLLr leukemias have very few cooperating mutations [44,45] but drastically alter the chromatin of the cell and so provide a useful model for aberrant chromatin changes in human disease. MLL-AF4 has been shown to drive gene expression by recruiting the H3 lysine 79 methyltransferase Disruptor of telomeric silencing 1-like (DOT1L) complex and increasing H3 lysine 79 di and trimethylation (H3K79me2/3) at gene targets [46]. Although traditionally H3K79me2 has been associated with transcription elongation, we discovered that inhibition of DOT1L in an MLL-AF4 ALL cell line disrupts enhancer–promoter interactions specifically at H3K79me2/3 marked enhancers (or KEEs) and is associated with a loss of H3K27 acetylation [7]. This led us to propose a model where H3K79me2/3 is essential for maintaining an open chromatin state and promoting transcription factor binding to drive enhancer activity at a subset of genes [7].

In more recent work, we found that MLL-AF4 itself as well as complex components such as the facilitates chromatin transcription (FACT) and RNA polymerase II (RNAPII)–associated factor 1 (PAF1) complexes are essential for maintaining enhancer–promoter interactions as well as eRNA transcription at key oncogenic enhancers ([47] and Figure 1d). Loss of MLL-AF4, FACT or PAF1 in an MLL-AF4 ALL cell line resulted in large domains of decreased enhancer–promoter proximity (as measured by 3C), a dramatic loss of eRNA transcription and global changes in H3K27ac [47]. These results contrast with the more subtle effects on enhancer–promoter proximity observed when degrading BRD4 or mediator [37,38,47]. Interestingly, partial degradation of PAF1 in a multiple myeloma cell line had only subtle effects on eRNA transcription and no impact on H3K27ac, suggesting that the central role for PAF1 in enhancer function may be unique to MLL-AF4 leukemias [47]. With this in mind, it is worth discussing the mechanistic role of PAF1 in some more detail.

## Polymerase-associated factor 1–mediated transcription elongation and enhancer function

*In vitro* transcription studies are consistent with a positive role for the PAF1 complex (PAF1C) in promoting transcription, where it has the capability of enabling RNAPII transcription through a nucleosome substrate [48–50]. The combined cryo-EM structures of an activated RNAPII complex provide a model for this activity, where PAF1 sterically competes with the pausing factor negative elongation factor for binding to RNAPII and along with other elongation factors promotes productive transcription [49,50].

Although the *in vitro* data implicate PAF1C in directly stimulating RNAPII activity in the context of chromatin, previous *in vivo* studies of PAF1C function have reported contradictory results, where it could promote both activation and repression. More recent genetic association studies have strongly implicated PAF1C in gene activation, where mutations of the PAF1C component CTR9 are associated with an ~10-fold increase in acquiring a myeloid malignancy [51]. Interestingly, the mechanism underlying this observation appears to be that loss of CTR9 causes aberrant recruitment of the remaining PAF1C to a subset of genes, causing increased gene activation and increased expansion of hematopoietic stem cells (HSCs) [51].

Consistent with an *in vivo* role in gene activation, recent work suggests PAF1C binding is correlated with highly active enhancers [47,52]. However, as already mentioned, PAF1C binding at enhancers is not always functional, as reduction of PAF1C in multiple myeloma cells has little impact on enhancer activity [47]. Instead, it is possible that PAF1C activity at enhancers is particularly prominent in MLL-AF4 leukemias where its binding is highly enriched at enhancers due to its association with the MLL-AF4 complex. MLL-AF4 and PAF1C together help create abnormally large enhancers with high-density interactions [47], but a role for PAF1C more generally in enhancer activity has yet to be fully established.

## The potential problem of patient heterogeneity in drug treatment

While MLL fusion proteins are themselves undruggable, recent work has focused on targeting various components of the fusion protein complex to disrupt its activity. The menin protein is a key component of MLL fusion protein complexes essential for their function [53]. Recent exciting clinical trials have shown that the inhibitor revumenib, which directly targets the MLL–menin interaction, is a valid drug target in MLLr leukemia patients [54,55].

However, even among patients harboring MLLr mutations, the overall initial response rate to revumenib was 53%, with only 30% displaying partial or complete remission [54]. Despite the excitement these results have generated, they also highlight a problem in clinical care. Even when patients carry the same driver mutation, they may not always respond the same way to therapy. This heterogeneity of patient response makes it difficult to predict the efficacy of specific therapies in individual patients. Some of this heterogeneity is likely caused by diverse mutational landscapes among individual patients, but a complementary explanation is that the heterogeneity in response is also driven by differential gene expression profiles between patients. As a key regulator of gene expression, one source of this transcriptional heterogeneity may be patient-unique chromatin environments, that is, epigenetic heterogeneity.

Despite carrying a very strong driver mutation, individual patients with MLLr ALL often show distinct expression profiles from each other. In particular, despite the fact that *MLL-AF4* infant ALL displays very few cooperating mutations [45], gene expression profiling can subset *MLL-AF4* infant ALL into distinct subgroups [56–58].

**Figure 2:**
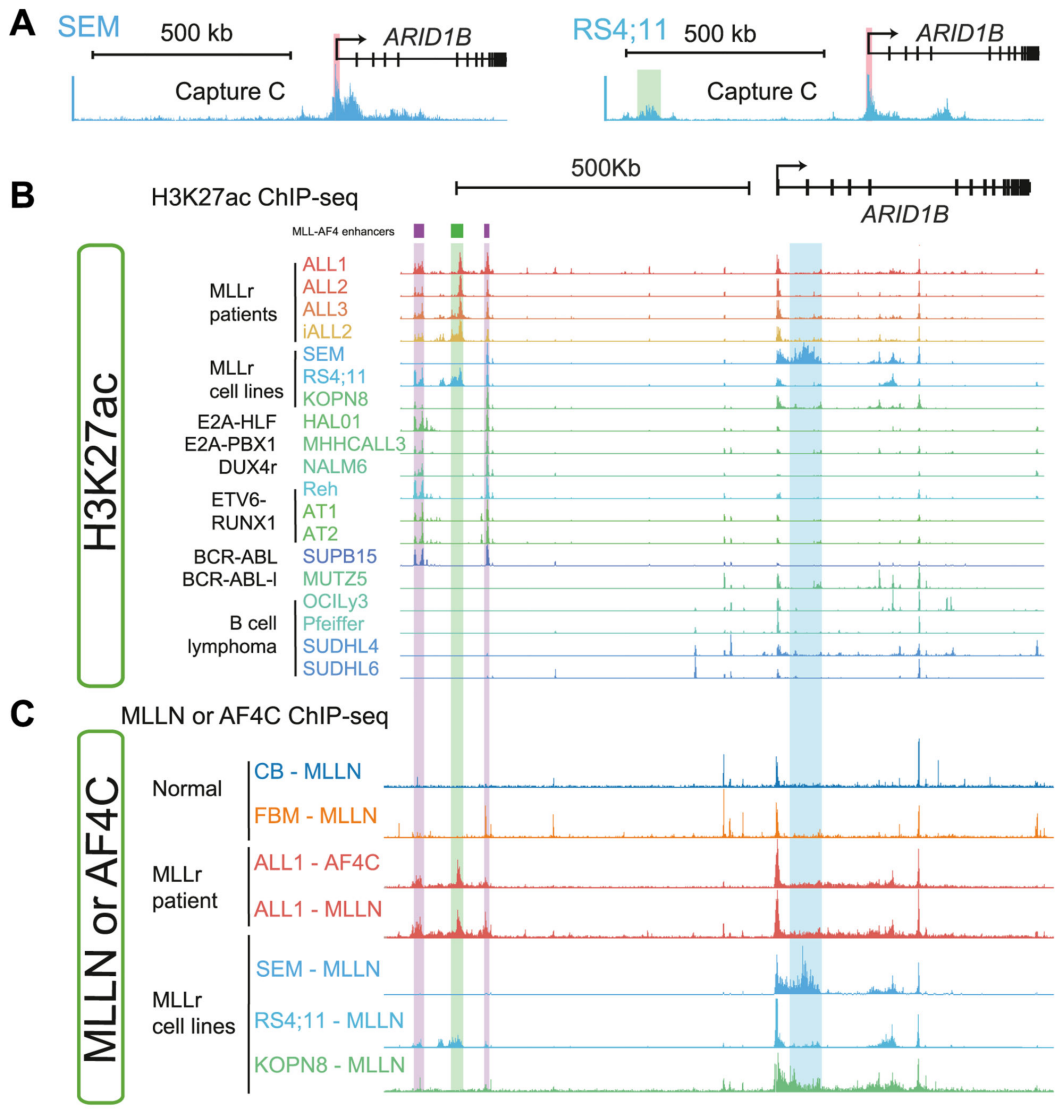
Novel enhancers in MLL-AF4 lekemia. **(a)** Capture C showing the frequency of interactions of DNA with the *ARID1B* promoter in two MLL-AF4 cell lines: SEM and RS4;11. The Capture viewpoint is shown by a red bar. The RS4;11 *ARID1B* upstream enhancer is highlighted by a green shaded bar. Data adapted from Ref.[7] **(b)** H3K27ac ChIP-seq at *ARID1B* in MLL-AF4 patient samples (childhood ALL1–3, infant ALL2) and the cell lines indicated. Enhancers are marked as found across multiple ALL samples (purple bar), unique to MLL-AF4 samples (green bar), or found in two or less MLL-AF4 samples (blue bar). Except for the SEM and KOPN8 unique enhancer in the gene body of *ARID1B*, enhancers are as called in Ref. [47]. Data and analysis are adapted from Refs. [47,62]. **(c)** Antibodies recognizing either the N terminus of MLL (MLLN) or the C terminus of AF4 (AF4C) were used for ChIP-seq to identify sites of MLL-AF4 binding (as in Ref. [47]) at *ARID1B* in normal cells (CB and FBM), an MLL-AF4 patient sample (childhood ALL1) or three MLLr cell lines (SEM, RS4;11 and KOPN8).

As an example, the *ARID1B* gene is a direct target of MLL-AF4 regulation [7,59], but expression of this gene can vary substantially between studies and between individual patients [59]. Interestingly, we found that *ARID1B* has two distinct enhancer profiles, as revealed by 3C studies in two different MLL-AF4 cell lines (Figure 2a and [7]). It appears that *ARID1B* expression can be driven by a large intragenic enhancer in the first intron of the gene, as seen in the MLL-AF4 cell line SEM and less so in the MLL-ENL KOPN8 cell line (Figure 2a,b). As measured by H3K27ac levels and MLL binding, this enhancer appears to be mostly absent in the MLL-AF4 RS4;11 cell line as well as in several MLL-AF4 patient cells (Figure 2b,c). Conversely, RS4;11 cells instead contain an intergenic enhancer about 500 kb upstream from the promoter (Figure 2a) that is also active in multiple MLL-AF4 patient samples (Figure 2b) but not in SEM or KOPN8 cells or in cell lines representing other leukemia subtypes (Figure 2b).

Enhancer activity correlates well with MLL-AF4 binding in patient cells or cell lines (Figure 2c), but none of these enhancers appears to be active in normal cord blood (CB) or fetal bone marrow cells (FBM; Figure 2c). Overall, this one example shows that an enhancer profile at specific genes can differ quite significantly between different patients, even though they carry the same MLLr mutation. The enhancer profile may also be unique compared with normal cells, together suggesting that one driver mutation may not only drive aberrant enhancer activity in a group of patients but may also drive differential enhancer profiles between different individual patients.

## Future perspectives

Much is still unknown about how enhancers function in normal cells and how this activity is disrupted in disease. Can MLLr ALL function as a paradigm for aberrant enhancer activity in disease? MLL-AF4-driven enhancers are abnormally large compared with normal enhancers, including those that exist in other ALLs [47]. The observation that PAF1C contributes to the activity of these MLL-AF4 enhancers but has only a minor effect at enhancers in the context of multiple myeloma suggests that the size and density of these enhancer–promoter interactions might be something specific to the MLL-AF4 complex itself. That is, it could be that PAF1C displays some level of hyperactivity in the context of the MLL-AF4 complex. Further work exploring this will require the use of degron lines for protein complex components of PAF1C or FACT in additional cellular contexts, including in non-MLLr ALL.

There appears to be some evidence for differential enhancer usage between MLL-AF4 patients, but it is unclear how extensive this is. Could aberrant changes in chromatin profiles drive unique expression profiles in individual patients, perhaps contributing to heterogeneity in drug response? To really understand this, we need to better understand the range of enhancer usage in individual MLL-AF4 patients and in other leukemia subtypes and how the activity of these enhancers relates to normal tissues. Emerging technologies suggest that there may be a place for using single-cell and multiplex profiling approaches to identify the full range of differential enhancer usage in normal tissues [60,61]. There are technical challenges that would need to be overcome to use these technologies in patient samples, but this could allow us to determine if individual patients display unique enhancer profiles. The long-term hope is that if patient heterogeneity leads to differential therapeutic response, and patient heterogeneity is driven by chromatin/enhancer differences, one exciting possibility is that chromatin profiles could eventually be used to predict patient responses.

## Data Availability

No new data were used for the research described in the article.
